# First report of complete chloroplast genome sequence of monotypic genus *Astilboides* (Saxifragaceae) in North East Asia

**DOI:** 10.1080/23802359.2020.1824595

**Published:** 2020-10-21

**Authors:** Young-Ho Ha, Hee-Young Gil, Dong-Kap Kim

**Affiliations:** a Division of Forest Biodiversity, Korea National Arboretum, Pocheon, Republic of Korea; b Department of Life Sciences, Gachon University, Seongnam, Republic of Korea; c DMZ Botanic Garden, Korea National Arboretum, Yanggu, Republic of Korea

**Keywords:** Chloroplast genome, *Astilboides tabularis*, Saxifragaceae, monotypic genus

## Abstract

The complete chloroplast genome sequence of *Astilboides tabularis*, one of endemic species of Eastern Asia, was determined. The chloroplast genome was 157,147 bp in length with large single-copy (87,703 bp), small single-copy (18,268 bp) and a pair of inverted repeats (25,588 bp). In total, 131 genes were encoded, including 86 protein-coding genes, 37 tRNA genes, and eight rRNA genes. The phylogenetic analysis using concatenated 77 protein-coding genes of 15 species chloroplast genome revealed that *A. tabularis* was sister to the clade containing *Bergenia*, *Oresitrophe*, and *Mukdenia*.

*Astiboides tubularis* (Hemsley) Engler (Saxifragaceae) is the only member of the genus *Astiboides* and is endemic to North East Asia. This monotypic genus is similar with *Rodgersia* A. Gray, but different by having large simple leaf and panicle inflorescence (Jintang and Cullen 2001; Lee 2003). This species is distributed in the regions of north eastern China (i.e. Jilin and Liaoning) and the Korean Peninsula (Lee 1996; Jintang and Cullen 2001; Yoon et al. 2015). In South Korea, *A. tabularis* was designated as an endangered and rare species and protected by multiple laws, such as Wildlife Protection and Management Act by the Ministry of Environment and Act on the Creation and Furtherance of Arboretums and Gardens by the Korea Forest Service (Lee 2009; Kim 2014). Furthermore, *A. tabularis* is an economically important plant resource as an edible and medicinal plant (Chang et al. 2005; Yang et al. 2020). In addition, its gigantic leaf and inflorescence have horticultural and ornamental values (Cho et al. 2016). Therefore, it is important to examine its genetic diversity and structure to conserve genetic resources of this species. As a first attempt, we report complete chloroplast (CP) genome sequence of *A. tabularis*.

Fresh leaves of *A. tabularis* were sampled from the living collection (KNKB 2020-15) of Korea National Arboretum, originally collected from Gangwon-do Province (37°29′ N 128°38′ E). Total genomic DNA was isolated using the DNeasy Plant Mini Kit followed by the manufacturer’s protocol (Qiagen Inc., Valencia, CA). Extracted high-quality gDNA was sequenced by using Illumina MiSeq (Illumina Inc., San Diego, CA) platform with 550 bp insert size. Paired-end reads were trimmed in Geneious R v. 10.2.6 program (Biomatters Ltd., Auckland, New Zealand). To construct the CP genome of *A. tabularis*, *Bergenia scopulosa* (NC_036061) was used as a reference. The chloroplast genome of *A. tabularis* was annotated by using the Dual Organellar GenoMe Annotator (DOGMA; Wyman et al. 2004) and tRNAscan-SE (Schattner et al. 2005). Phylogenetic tree including 13 species of Saxifragaceae was constructed based on concatenated 77 chloroplast protein coding genes using IQ-TREE v.1.6.8 (Nguyen et al. 2015) with 1000 bootstrap replications (Figure 1).

**Figure 1.**
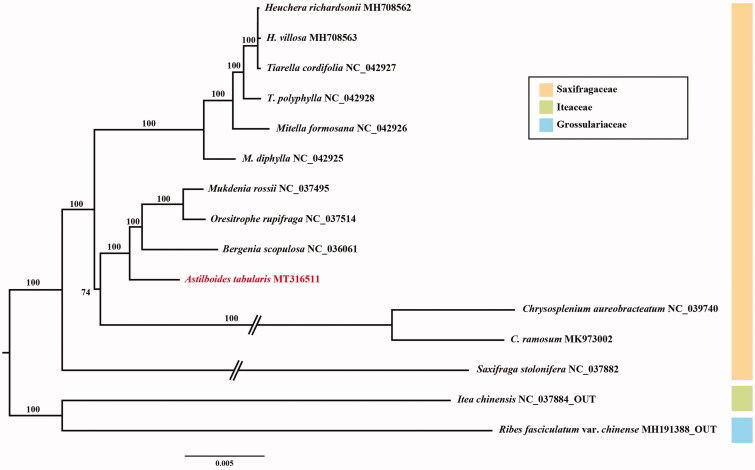
Maximum-likelihood tree based on 77 protein-coding genes from 13 representative species of Saxifragaceae. Bootstrap support values are shown at the branches.

The CP genome of *A. tabularis* (MT316511) has a typical quadripartite structure (157,147 bp) including large single copy (LSC; 87,703 bp), small single copy (SSC; 18,268 bp) and a pair of inverted repeats (IRa and IRb; 25,588 bp). The chloroplast genome contained 131 genes including 86 protein-coding genes, 37 tRNA genes, and eight rRNA genes. The phylogenetic analysis of Saxifragaceae showed that *A. tabularis* is sister with the clade containing *Bergenia*, *Oresitrophe*, and *Mukdenia* (bootstrap value = 100%). Further work on details is necessary to elucidate phylogenetic relationships of the monotypic genus, *Astilboides*, and other major lineages of Saxifragaceae.

## Data Availability

The data that support the findings of this study are openly available in GenBank of NCBI at https://www.ncbi.nlm.nih.gov, reference number MT316511.
